# Spigelian Hernia: The Unseen Among the Usual

**DOI:** 10.7759/cureus.106759

**Published:** 2026-04-09

**Authors:** Minesh Sindhal, Priyanka Aanandaka, Divyeshkumar N Parmar, Mohith VS, Anand Kumar

**Affiliations:** 1 General Surgery, All India Institute of Medical Sciences, Rajkot, Rajkot, IND

**Keywords:** groin swelling, inguinal hernia mimic, laparoscopic hernia repair, spigelian hernia, tapp repair

## Abstract

Not every groin swelling is what it appears to be. An uncommon presentation of a common symptom can challenge even routine clinical assumptions. We report a 34-year-old female with a long-standing right groin swelling, clinically indistinguishable from an inguinal hernia. Despite supportive examination and imaging, laparoscopy identified an intact inguinal canal and instead identified a small defect along the lateral border of the rectus abdominis, consistent with a Spigelian hernia. A laparoscopic transabdominal preperitoneal (TAPP) repair with mesh placement was performed successfully, and recovery was uneventful. This case underscores a key surgical lesson: rare hernias can convincingly mimic common ones. In such scenarios, laparoscopy serves as both a decisive diagnostic tool and a definitive means of treatment.

## Introduction

Groin swellings in women are often approached with a quiet diagnostic certainty - most will prove to be inguinal or femoral, and clinical experience tends to reinforce this expectation [1]. However, it is precisely within such familiarity that uncommon pathology finds room to conceal itself. Spigelian hernia, an infrequent and often under-recognized defect of the anterior abdominal wall, occupies this diagnostic blind spot, representing a very small proportion of all abdominal wall hernias, and rarely declares itself in a manner that raises early suspicion [2].

The present case challenged that comfort. A 34-year-old female presented with a right groin swelling of long duration, exhibiting every clinical hallmark of an inguinal hernia - reducibility, a well-defined cough impulse, and a slow, progressive course. Neither examination nor imaging provided a compelling reason to question this assumption. The diagnosis appeared settled well before the patient reached the operating table.

It was only under the laparoscopic view that the narrative shifted. The inguinal canal, anticipated to harbor the defect, was found to be intact. Instead, a small but definitive fascial breach was identified along the lateral edge of the rectus abdominis, positioned superior to the deep inguinal ring - an anatomical location characteristic of a Spigelian hernia. The proximity of this defect to the groin, coupled with its ability to reproduce classical clinical signs, rendered it a near-perfect mimic.

A transabdominal preperitoneal approach offered both clarity and control - permitting precise dissection, full appreciation of the defect, and a well-positioned mesh repair with generous overlap. The procedure was completed without difficulty, and recovery was uneventful.

What this case ultimately reinforces is not merely the rarity of Spigelian hernia but the limits of diagnostic familiarity. In such situations, laparoscopy becomes more than a technique - it becomes an extension of surgical judgment, revealing what clinical reasoning alone may overlook.

## Case presentation

A 34-year-old married female presented with a swelling in the right groin region for 2.5 years. The swelling was insidious in onset and gradually progressive. Over the preceding three months, she experienced increasing pain associated with the swelling, characterized as dull and dragging, exacerbated by straining, coughing, and physical exertion. She worked as a maid and reported a history of frequent heavy lifting. There was no history of chronic cough, constipation, urinary symptoms, or prior abdominal surgeries. She had no known comorbidities. Her body mass index was 26 kg/m².

Examination revealed a 3 × 3 cm swelling in the right inguinal region. The swelling was globular, soft in consistency, and had ill-defined margins. A positive cough impulse was elicited, and the swelling appeared reducible on lying down. There were no overlying skin changes or signs of inflammation. The contralateral groin was normal, and no lymphadenopathy was noted. Based on clinical findings, a provisional diagnosis of right inguinal hernia was made.

Routine blood work did not demonstrate any derangements. Ultrasonography of the groin suggested an inguinal hernia. However, in view of the chronicity of the condition, a contrast-enhanced computed tomography (CECT) scan was performed. CECT demonstrated a defect in the anterior abdominal wall in the right lower abdomen, more suggestive of a ventral hernia in the inguinal region with omentum as content and fibrotic changes with a defect size of 10-11 mm. The patient was planned for laparoscopic hernia repair.

Laparoscopic exploration was performed using a standard three-port technique under general anaesthesia, employing a transabdominal preperitoneal (TAPP) approach.

Initial inspection of the inguinal region did not reveal any direct or indirect inguinal hernia. Instead, a small defect measuring approximately 1 × 1 cm was identified along the lateral border of the rectus abdominis muscle, approximately 5 cm superior to the right deep inguinal ring.

**Figure 1:**
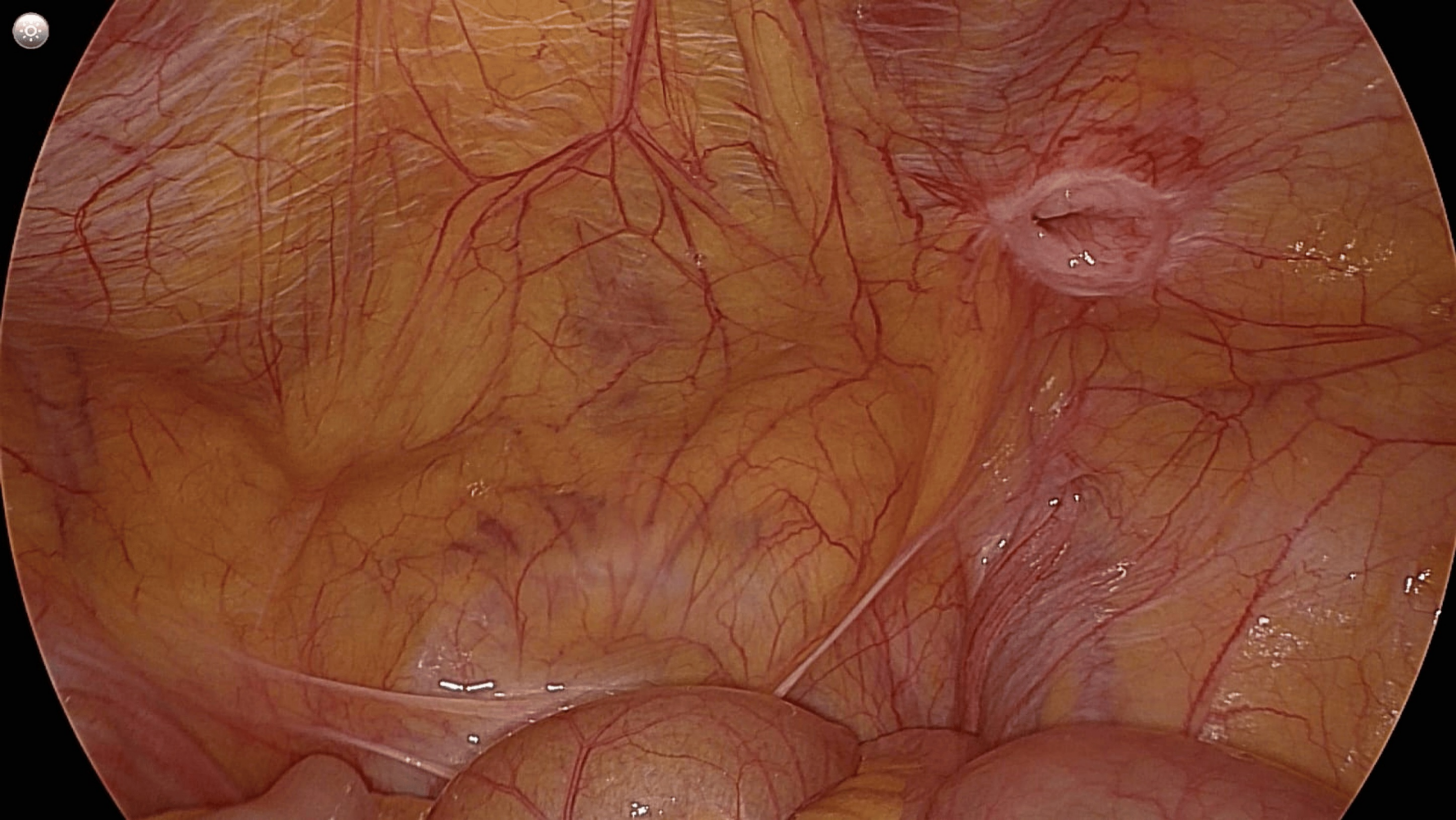
Laparoscopic view of the anterior abdominal wall demonstrating a Spigelian hernia defect located along the semilunar line, characteried by a well-circumscribed fascial defect with clearly defined margins.

Figure 1 presents the laparoscopic view of the anterior abdominal wall demonstrating a Spigelian hernia defect located along the semilunar line, characterised by a well-circumscribed fascial defect with clearly defined margins. A peritoneal sac is seen protruding through the defect.

This location corresponded to the Spigelian fascia within the Spigelian belt, confirming the diagnosis of a Spigelian hernia. No hernial contents were visualized within the sac at the time of surgery, likely due to spontaneous reduction following pneumo-peritoneum creation.

A peritoneal incision was made approximately 5 cm above the defect to create a peritoneal flap. Careful preperitoneal dissection was carried out: medially, dissection was extended beyond the midline; laterally, 5 cm beyond the external iliac vessels; and inferiorly, approximately 2 cm below Cooper's ligament.

After adequate space creation, a 15 × 15 cm polypropylene mesh was placed into the preperitoneal space, ensuring wide overlap of the defect in all directions. The mesh was positioned without tension and flattened appropriately.

**Figure 2:**
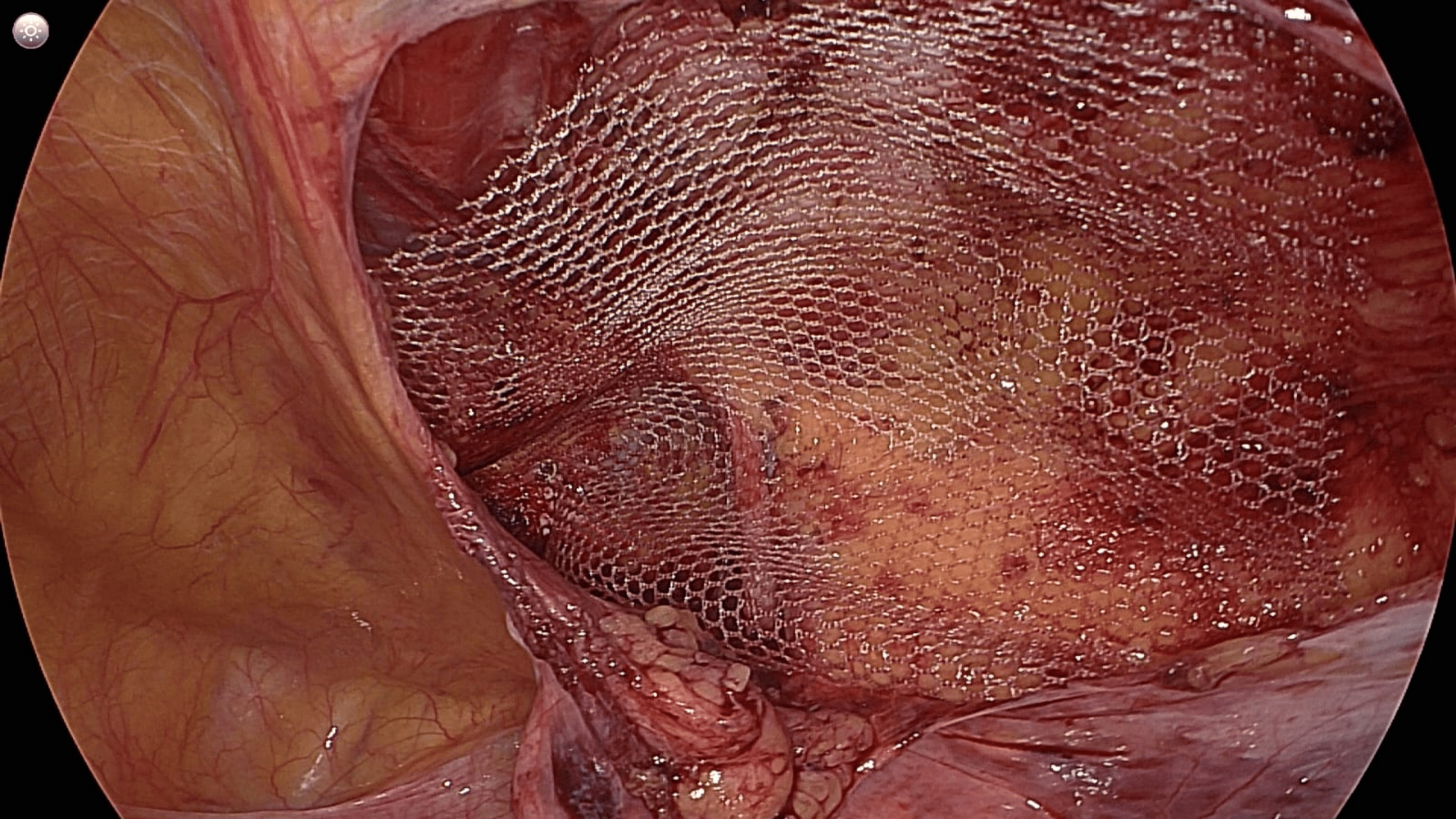
A prosthetic mesh is seen in-situ, adequately covering the defect with good circumferential overlap.

In Figure 2, a prosthetic mesh is seen in-situ, adequately covering the defect with good circumferential overlap. The mesh appears in close approximation with the anterior abdominal wall without folding.

The peritoneal flap was then closed to completely cover the mesh. Hemostasis was secured, and the procedure was completed without complications.

The patient had an uneventful recovery. She was ambulated on the same day, and an oral diet was initiated within six hours. Postoperative pain was minimal. She was discharged on postoperative day four.

## Discussion

Spigelian hernia is a rare anterior abdominal wall defect, accounting for less than 2% of hernias, and is frequently overlooked in routine clinical evaluation [3]. The majority of these defects occur within the so-called Spigelian belt, where the posterior rectus sheath is deficient, creating a potential site of weakness. Its interparietal course often prevents obvious external swelling, making diagnosis challenging.

When located in the lower abdomen, it may closely mimic an inguinal hernia due to its proximity to the deep inguinal ring. Typical clinical findings, including reducibility and an expansile impulse on coughing, may be present, leading to diagnostic confusion, as seen in this case [4].

Imaging modalities have limitations. Ultrasonography is operator-dependent, and although computed tomography provides better anatomical detail, it may not reliably differentiate small or reducible defects [5]. As seen in this case, imaging suggested pathology but lacked definitive anatomical clarity.

Laparoscopy plays a key role in such cases by allowing direct visualization and accurate identification of the defect. The TAPP approach enables both diagnosis and effective mesh repair with adequate coverage [6]. Furthermore, the laparoscopic approach allows simultaneous assessment of the inguinal region, ensuring that no coexisting pathology is overlooked.

This case emphasizes the need to consider rare hernias in atypical presentations and highlights laparoscopy as a valuable tool for both diagnosis and management.

## Conclusions

A Spigelian hernia is a distinctly rare abdominal wall defect that often remains outside routine clinical consideration. Its infrequency, coupled with its ability to closely resemble more common groin pathologies, makes it particularly prone to misdiagnosis. This case highlights how an uncommon condition can present with entirely familiar clinical features, potentially misleading even experienced clinicians. Recognition of such rare entities is essential, especially when findings do not fully align. Maintaining a broader diagnostic perspective is crucial. Ultimately, a Spigelian hernia serves as a reminder that rarity does not preclude relevance and that careful evaluation is key to accurate diagnosis and appropriate surgical management.

## References

[1] Fitzgibbons RJ Jr, Forse RA (2015). Groin hernias in adults. N Engl J Med.

[2] Skandalakis PN, Zoras O, Skandalakis JE, Mirilas P (2006). Spigelian hernia: surgical anatomy, embryology, and technique of repair. Am Surg.

[3] Hanzalova I, Schäfer M, Demartines N, Clerc D (2022). Spigelian hernia: current approaches to surgical treatment - a review. Hernia.

[4] Clark CR, Kelly ML, Palamuthusingam P (2024). Spigelian hernia: a multi-site review of operative outcomes of surgical repair in the adult population. Hernia.

[5] Mabrouk A, Fatnassi O, Boukhchim M, Jedidi L, Lahouel SB, Karmous N, Moussa MB (2025). Spigelian hernia: diagnostic challenges and therapeutic approaches: report of fourteen clinical cases (version 1; peer review: 2 not approved). F1000Res.

[6] Ding D, Wang Y, You J, Wei J, Liang R, Wei B (2025). Laparoscopic repair for Spigelian-inguinal hernia complex: a case report and review of literature. Int J Surg Case Rep.

